# Biocatalytic production of the antibiotic aurachin D in *Escherichia coli*

**DOI:** 10.1186/s13568-022-01478-8

**Published:** 2022-11-03

**Authors:** Sebastian Kruth, Lina Schibajew, Markus Nett

**Affiliations:** Department of Biochemical and Chemical Engineering, TU Dortmund University, Dortmund, Germany

**Keywords:** Aurachin, Bicistronic design, *Escherichia coli*, Farnesyltransferase, Heterologous expression, Membrane protein

## Abstract

**Abstract:**

Aurachin D is a potent inhibitor of cytochrome *bd* oxidases, which are potential targets in the treatment of infectious diseases. In this study, our aim was to improve the biocatalytic production of aurachin D from a quinolone precursor molecule with recombinant *Escherichia coli* cells expressing the biosynthesis enzyme AuaA. In order to achieve a high-level production of this membrane-bound farnesyltransferase in *E. coli*, the expression of the *auaA* gene was translationally coupled to an upstream cistron in accordance with a bicistronic design (BCD) strategy. Screening of various BCD elements led to the identification of optimized *auaA* expression cassettes, which increased the aurachin D titer in *E. coli* up to 29-fold in comparison to T7-mediated expression. This titer could be further raised by codon optimization of *auaA* and by introducing the mevalonate pathway into the production strain. The latter measure was intended to improve the availability of farnesyl pyrophosphate, which is needed as a cosubstrate for the AuaA-catalyzed reaction. In sum, the described efforts resulted in a strain producing aurachin D with a titer that is 424 times higher than that obtained with the original, non-optimized expression host.

**Graphical Abstract:**

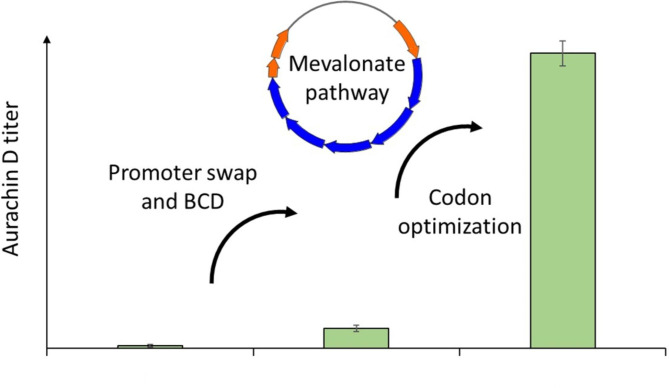

**Supplementary Information:**

The online version contains supplementary material available at 10.1186/s13568-022-01478-8.

**Key points**

- the expression of the *auaA* gene is a bottleneck for aurachin production in *E. coli*.
- *auaA* expression can be improved using a bicistronic design architecture.
- introduction of the mevalonate pathway into *E. coli* stimulates aurachin production.

## Introduction

The aurachins are prenylated quinolone antibiotics, which are naturally produced by myxobacteria of the genus *Stigmatella* (Kunze et al. 1987; Höfle and Irschik 2008) and by some actinobacterial strains (Kitagawa and Tamura 2008; Nachtigall et al. 2010; Zhang et al. 2017). Since their identification as inhibitors of electron transport chains (Meunier et al. 1995), these natural products have become important tool compounds. An illustrative example is aurachin D, which provided important structural and functional insights into NADH-ubiquinone oxidoreductases and cytochrome *bd* quinol:dioxygen oxidases, respectively (Ito et al. 2017; Masuya et al. 2020; Grauel et al. 2021). The aurachins have also attracted interest in drug development as inhibitors of the bacterial respiratory chain (Lu et al. 2015). For this reason, methods for their chemical synthesis were sought. Several of these efforts were directed toward the preparation of the model compound aurachin D. In the reported syntheses, the installation of the farnesyl substituent always took place before the quinolone heterocycle was formed (Dejon and Speicher 2013; Li et al. 2013; Enomoto et al. 2014; Radloff et al. 2021). Attempts to introduce the isoprenoid side chain chemically after the assembly of the quinolone scaffold were not successful (Dejon and Speicher 2013). This is regrettable because 4-quinolones can be easily prepared by organic synthesis (Ghosh and Das 2019) and the possibility to convert such molecules into aurachins would allow the production of large compound libraries for structure-activity relationship studies. The use of an enzyme, which carries out the required transformation with high regioselectivity, could offer a solution to this problem.

**Fig. 1:**
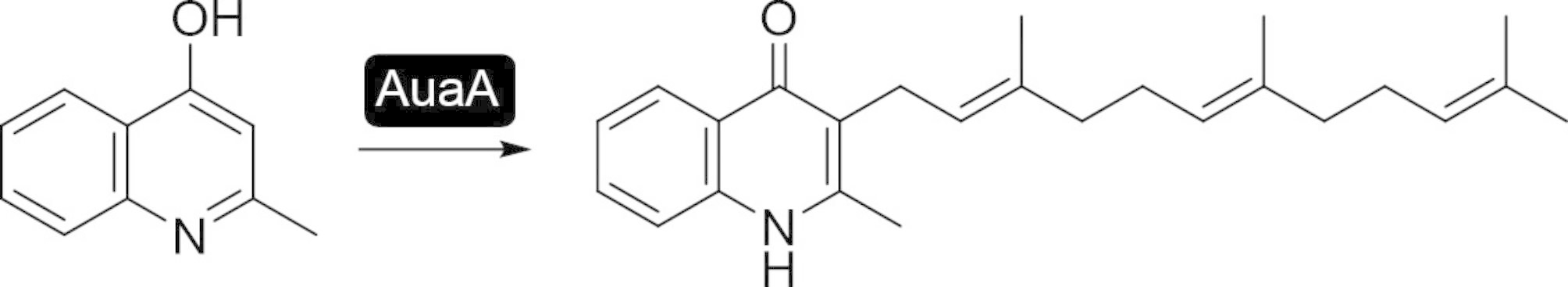
Biosynthetic conversion of 4-hydroxy-2-methyl-quinoline (HMQ) into aurachin D by the membrane-bound farnesyltransferase AuaA

In aurachin biosynthesis, the membrane-bound protein AuaA is responsible for the transfer of farnesyl diphosphate (FPP) onto 4-hydroxy-2-methyl-quinoline (HMQ), thus producing aurachin D (Fig. 1) (Sandmann et al. 2007; Stec et al. 2011). Biochemical studies revealed that AuaA is capable to process a variety of unnatural substrates, albeit with reduced activity in most cases (Stec et al. 2011). The use of native aurachin producers as whole cell biocatalysts is feasible for the generation of analogs, but it leads to compound mixtures with the HMQ-derived metabolites as the dominant products (Sester et al. 2020). Although it is in theory possible to abolish HMQ production in a native producer by targeted gene inactivation and to produce the desired derivatives of aurachin D by mutasynthesis, this approach will also result in a complex product spectrum due to subsequent biosynthetic transformations of the AuaA product (Pistorius et al. 2011). To circumvent an extensive downstream processing, it seems advisable to carry out the prenylation reaction by biotransformation (Winand et al. 2021) using a heterologous host expressing *auaA*. An in vitro prenylation with purified AuaA would be another alternative, but this option requires the addition of FPP as a cosubstrate. To keep the costs low, we decided to conduct the AuaA reaction with whole cells, which are capable of FPP biosynthesis.

The bacterium *Escherichia coli* was chosen as host for the planned biotransformation for various reasons. First of all, the membrane protein AuaA had already been functionally expressed in *E. coli* for in vitro studies, even though the in vivo production of aurachins had not been reported in the corresponding study (Stec et al. 2021). In addition, *E. coli* possesses several attractive traits, such as fast growth and its ease of genetic manipulation (Zhang et al. 2019). Another advantage is the minimal secondary metabolite background of *E. coli* cells, which facilitates the recovery of heterologously produced compounds (Ma et al. 2021).

In this study, we report the construction of an *E. coli* strain that is adapted for the expression of AuaA and can also supply sufficient FPP for the AuaA-catalyzed prenylation reaction. Upon feeding of HMQ, this strain generates aurachin D with a titer that exceeds all previously described producer organisms.

## Materials and methods

### Strains, plasmids, media, and growth conditions

The bacterial strains and plasmids used in this study as well as their relevant characteristics are listed in Table 1. The native aurachin producer *Stigmatella erecta* Pd e32 was grown at 30 °C on agar plates containing modified Zein medium (Sester et al. 2020). *E. coli* strains were routinely cultivated in standard lysogeny broth (LB) medium (Carl Roth) at 37 °C and 200 rpm, unless they possessed an *auaA* expression plasmid. In such a case, the respective strain was grown at 30 °C. For maintenance of plasmids, antibiotics were added to the media at the following concentrations: 100 µg mL^− 1^ ampicillin, 50 µg mL^− 1^ kanamycin, and chloramphenicol 30 µg mL^− 1^. The growth of *E. coli* cultures was followed by measuring the optical density at 600 nm (OD_600_).

**Table 1:** Strains and plasmids used in this study

Strain or plasmid	Relevant characteristics	Source or reference
*E. coli* strains		
DH5α	F– φ80*lacZ*Δ M15 Δ (*lacZYA-argF*) *U169 recA1 endA1 hsdR17* (rK– mK+) *phoA supE44* λ- *thi–1 gyrA96 relA1*	Invitrogen
TOP10	F-*mcrA* Δ( *mrr-hsd*RMS-*mcr*BC) Φ80*Lac*ZΔM15 Δ *Lac*X74 *rec*A1 *ara*D139 Δ( *araleu*) 7697 *gal*U *gal*K *rps*L (StrR) *end*A1 *nup*G	Invitrogen
BL21(DE3)	F − *ompT hsdSB*(r_B_− m_B_−) *gal dcm* (DE3)	Merck KGaA
*S. erecta* strains		
PD e32	natural aurachin producer	DSMZ
Plasmids		
pJet1.2/blunt	*amp*^R^, *E. coli* cloning vector (pMB1 ori, *eco47IR*, P_*lac*UV5_, P_T7_)	Thermo Fisher
pET-28a(+)	*kan*^R^, T7-based *E. coli* expression vector (pBR322 ori, f1 ori, *lacI*, *rob*, P_T7_, *lac* operator, His-Tag, T7 terminator)	Novagen
pSK39	pET-28a(+) derivative for *auaA* expression	This study
pFAB3913	*kan*^R^, *E. coli* BCD-based expression vector (p15A ori, P_trc*_BCD9, *mRFP1*)	Mutalik et al. 2013
pWaldo-GFPe-SH1446	*kan*^R^, pWaldo-GFPe derivative encoding the POT family transporter SH1446 with a C-terminal TEV-GFP-His-Tag (pBR322 ori, f1 ori, *lacI*, *rob*, P_T7_, *lac* operator, SH1446, T7 terminator)	Minhas et al. 2018
pSK40	pFAB3913 derivative containing the TEV-folding reporter *gfp*-His-Tag instead of *mRFP1*	This study
pSK41	pET-28a(+) derivative harboring the expression cassette of pSK41 instead of the T7 expression cassette (P_trc*_ BCD9, TEV-folding reporter *gfp*-His-Tag)	This study
pSK42	pSK41 derivative without *lacI*	This study
pSK43	pSK44 derivative without BCD9 element for monocistronic expression of *auaA*	This study
pSK44	pSK42 derivative for expression of *auaA* fused to TEV-folding reporter *gfp*-His-Tag	This study
pSK45	pSK42 derivative for expression of *auaA*_Se fused to TEV-folding reporter *gfp*-His-Tag	This study
pSK46	pSK42 derivative for expression of *auaA*_Sa fused to TEV-folding reporter *gfp*-His-Tag	This study
pJBEI-2997	*cm*^R^, *E. coli* plasmid harboring the mevalonate pathway (p15A ori, *lacI*, P_*lac*UV5_, *atoB*, HMGS, tHMGR, MK, PMK, PMD, *idi*, *ispA*)	Peralta-Yahya et al. 2011

*amp*^R^ ampicillin resistance, *kan*^*R*^ kanamycin resistance, *cm*^R^ chloramphenicol resistance

For the recombinant production of aurachin D, *E. coli* strains were grown in 25 mL of terrific broth (TB) medium (Tartoff and Hobbs 1987), which had been supplemented with 0.04% (v/v) glycerol and 0.003% (w/v) HMQ. The production cultures were inoculated to an OD_600_ of 0.1 and, subsequently, cultivated at 30 °C and 200 rpm for 24 h. If necessary, the cultures were induced with 1 mM isopropyl *β*-d-1-thiogalactopyranoside (IPTG) after they reached an OD_600_ of 1. To induce the expression of the mevalonate biosynthesis genes from pJBEI-2997 (Peralta-Yahya et al. 2011), IPTG was added to a final concentration of 0.025 mM.

### General DNA methods

Genomic DNA of *S. erecta* Pd e32 was extracted from an agar culture using the DNeasy Blood & Tissue Kit (Qiagen). Plasmid DNA was isolated from *E. coli* strains with the NucleoSpin Plasmid (NoLid) Kit (Macherey-Nagel). PCR amplifications were carried out with Phusion Hot Start II DNA-Polymerase (ThermoFisher) except for colony PCRs, which were performed with DreamTaq DNA Polymerase (Thermo Fisher Scientific). DNA fragments were directly isolated after PCR or extracted from agarose gels using the NucleoSpin Gel and PCR Clean-up Kit (Macherey-Nagel). Plasmids were digested with FastDigest restriction enzymes (Thermo Fisher Scientific). Ligations were performed with T4 DNA ligase (Thermo Fisher Scientific). For Gibson assembly, the GeneArt Gibson Assembly HiFi Master Mix (Invitrogen, Thermo Fisher Scientific) was used. Transformation of *E. coli* DH5α, TOP10 and BL21 (DE3) followed the protocol of Hanahan (1983). Subcloning was generally performed in *E. coli* DH5α or TOP10.

### Construction of expression plasmids

The aurachin (*aua*) operon was amplified by PCR from genomic DNA of *S. erecta* Pd e32 using the primers SK40 (5’-ATCTGGCGCAGCATGATGAG-3’) and SK57 (5’-CCAAGCACGGCTTCTTCAG-3’). The amplicon was ligated into pJet1.2/blunt to give pSK38. Subsequently, the *auaA* gene from *S. erecta* was cloned into pET-28a(+) by Gibson assembly (Gibson et al. 2009). For this, pET-28a(+) was digested with EcoRI and NcoI, while the insert was amplified by overhang PCR from pSK38 using the primers SK227 (5’-TTGTTTAACTTTAAGAAGGAGATATACCATGACCCTGCTCCAAGTG-3’) and SK228 (5’-TGTCGACGGAGCTCGAATTCTCACGAAGCGACGAAGAC-3’). The linearized pET-28a(+) and the PCR product were then assembled to the expression vector pSK39.

The construction of *auaA* expression plasmids featuring the constitutive promoter P_trc*_ started from the previously described pFAB3913, which already contained a BCD element (Mutalik et al. 2013). At first, the *mRFP1* gene on pFAB3913 was replaced with the folding reporter *gfp*. This was achieved by Gibson assembly. For this, pFAB3913 was linearized by PCR using the primers SK202 (5’-GGATCGGTTGTCGAGTAAG-3’) and SK203 (5’-CATTAGAAAGACTCCTCTGCATG-3’). The *gfp* insert was obtained by PCR using the primers SK204 (5’-GCAGAGGAGTCTTTCTAATGGAAAACCTGTACTTCCAGGG-3’) and SK205 (5’-CCTTACTCGACAACCGATCCGTTAGCAGCCGGATCT-3’) and the plasmid pWaldo-GFPe-SH1446 as a template (Minhas et al. 2018). The expression cassette of the assembled vector, pSK40, was excised by restriction digestion and introduced into the XhoI-BglII site of pET-28a(+) to construct pSK41. Inverse PCR with the 5’ phosphorylated primers SK206 (5’-GGTGATGTCGGCGATATAG-3’) and SK207 (5’-CTGGTTAGCAGAATGAATCAC-3’) was then used to remove the *lacI* gene on pSK41. The PCR product was circularized upon treatment with T4 DNA ligase, thus yielding pSK42. The plasmid pSK42 provided the backbone for all P_trc*_-driven expression vectors in this study.

Three variants of the *auaA* gene were independently cloned into pSK42. These variants included the native *auaA* gene from *S. erecta* Pd e32 (Genbank accession number ON677237), as well as alternative versions of this gene and its homolog from *S. aurantiaca* Sg a15, which had been codon and sequence optimized for expression in *E. coli*. The optimized sequences had been calculated with the software GeneOptimizer and synthesized by GeneArt (Invitrogen, Thermo Fisher Scientific). They are in the following referred to as *auaA*_Se and *auaA*_Sa. The sequences of *auaA*_Se and *auaA*_Sa were deposited in GenBank under accession numbers ON677238 and ON677239. Gibson assembly was used for the construction of the pSK42-derived expression plasmids. Briefly, pSK42 was linearized by PCR with primers SK208 (5’-TAGAAAGACTCCTCTGCATGA-3’) and SK209 (5’-GAAAACCTGTACTTCCAGGG-3’). The inserts were obtained by overhang PCR using the primers SK210 (5’-CATGCAGAGGAGTCTTTCTAATGACCCTGCTCCAAG-3’) and SK211 (5’-CCCTGGAAGTACAGGTTTTCCGAAGCGACGAAGACC-3’) for *auaA*, or SK212 (5’-CATGCAGAGGAGTCTTTCTAATGACCCTGC TGCAG-3’) and SK213 (5’-CCCTGGAAGTACAGGTTTTCGCTGGCAACAAAAACACG-3) for the amplification of *auaA*_Se and *auaA*_Sa. The linearized vector and the respective insert were joined by Gibson assembly to give pSK44 (*auaA*), pSK45 (*auaA*_Se), and pSK46 (*auaA*_Sa). The control plasmid for monocistronic expression of *auaA*, pSK43, was generated by inverse PCR with the 5’ phosphorylated primers SK231 (5’-ATGACCCTGCTCCAAGTGG-3’) and SK232 (5’-TGTTGATCTCCTTTTTAAGTGAACTTGG-3’) to delete the BCD9 element. The PCR product was circularized upon treatment with T4 DNA ligase to give pSK43.

### Construction of BCD libraries

In order to generate plasmids with varying BCD elements, pSK44, pSK45 and pSK46 were equipped with Eco31I restriction sites to enable a rapid replacement of the existing translational coupling unit via Golden Gate cloning (Engler et al. 2008). The Eco31I restriction sites were introduced by PCR using the primers SK217 (5’-TAATTAGGTCTCATGTTTCAGTACGAAAATTGCTTTCATTG-3’) and SK218 (5’-TAATTAGGTCTCTAATGACCCTGCTCCAAGTG-3’) for pSK44, or the primers SK217 and SK219 (5’-TAATTAGGTCTCTAATGACCCTGCTGCAGGTTG-3’) for pSK45 and pSK46, respectively. The Eco31I-digested PCR products were assembled one by one with the pooled annealed oligo pairs BG7291-BG7334 following a previously described protocol (Claassens et al. 2019). The ligation mixes were directly used for the transformation of *E. coli* BL21(DE3).

### Screening of the BCD library

All transformants were picked (*auaA*: 57 colonies, *auaA*_Se: 96 colonies, *auaA*_Sa: 30 colonies) and the corresponding colonies were individually used to inoculate the wells in sterile 96-well black, clear-bottom plates (Greiner Bio-One). Each well contained 200 µL LB medium and kanamycin. The plates were incubated in a microbioreactor (BioLector I, m2p-labs) at 30 °C and 950 rpm for 15 to 18 h. Microbial growth was monitored during the cultivation by scattered light measurements using a gain of 25. Furthermore, the formation of the AuaA-GFP fusion protein was followed online by fluorescence measurements using a gain of 85. After the incubation, 20 cultures were selected on the basis of their growth and fluorescence. To this end, the normalized fluorescence (n.f.) was calculated after 15 h as ratio of fluorescence intensity to scattering light intensity. The n.f. threshold values for selection were 1.2 (*auaA*), 1.8 (*auaA*_Se), and 1.7 (*auaA*_Sa), respectively. From every selected culture 16 µL were inoculated into 800 µL of fresh LB medium with kanamycin in a 48-well FlowerPlate (m2p-labs). These plates were incubated in the BioLector I at 30 °C and 1400 rpm for 24 h. Again, biomass formation and fluorescence were measured online. After 20 h of incubation, nine cultures were chosen featuring scattering light values > 190 and n.f. values > 1 for *auaA* and *auaA*_Se or > 1.8 for *auaA*_Sa.

### Quantification of aurachin D titers

For the quantification of aurachin D, 10 mL of each production culture was extracted three times with 15 mL ethyl acetate. The organic phases were collected and the solvent was evaporated. The residue was dissolved in 1 mL methanol, filtered and subjected to HPLC analysis. The latter was carried out on a Shimadzu Nexera Series LC 40 system equipped with a photodiode array detector and a Nucleodur 100-5 C18 ec column (250 × 4.6 mm, 5 μm, Macherey-Nagel). For the separation, a linear gradient of methanol in water supplemented with 0.1% (v/v) trifluoroacetic acid from 40 to 90% over 20 min was used. The flow rate was set to 1 mL min^− 1^. The aurachin D peak was identified by comparison with an authentic, commercial standard (AOBIOUS). Its absorption was measured at 236 nm and the concentration was calculated using a calibration curve of the aurachin D standard (Figure S1). All production cultures and quantification experiments were run in three technical replicates, if not stated otherwise.

## Results

**Expression of*auaA*in*E. coli*and generation of bicistronic design (BCD) vectors**.

Initially, the *auaA* gene was expressed in *E. coli* BL21(DE3) under the control of the T7 promoter. Although the expression strain was capable to farnesylate exogenously supplied HMQ, only a very low aurachin D titer of 40 µg L^− 1^ was achieved. One possible explanation for the weak performance was that the expression had not provided satisfactory amounts of active AuaA. The heterologous production of membrane-bound enzymes is known to be challenging. It can be limited by ribosome binding site (RBS) accessibility, the availability of chaperones and membrane translocation systems, as well as by toxicity issues resulting from the disruption of the natural membrane (Gialama et al. 2017). Often these problems can be overcome by fine-tuning the expression level using transcriptional and translational fusions (Schlegel et al. 2014). Since the attachment of solubility tags to membrane proteins can affect their structure and function (Marino et al. 2015), we decided to use transcriptional fusion as a means to improve the expression of *auaA*. For this purpose, we used the constitutive trc* promoter and implemented a bicistronic design (BCD), in which the start codon of the gene of interest is nearby or overlapping with the stop codon of an upstream cistron (Sun et al. 2021). The idea behind this architecture, which is naturally present in many bacterial operons, is that the translation of the leading cistron will additionally resolve downstream secondary structures in the mRNA due to the intrinsic helicase activity of ribosomes (Takyar et al. 2005). The reason for replacing the strong T7 promoter with the trc* promoter was that the latter had been reported to outperform the former for membrane protein production in conjunction with a BCD architecture (Claassens et al. 2019).

**Fig. 2:**
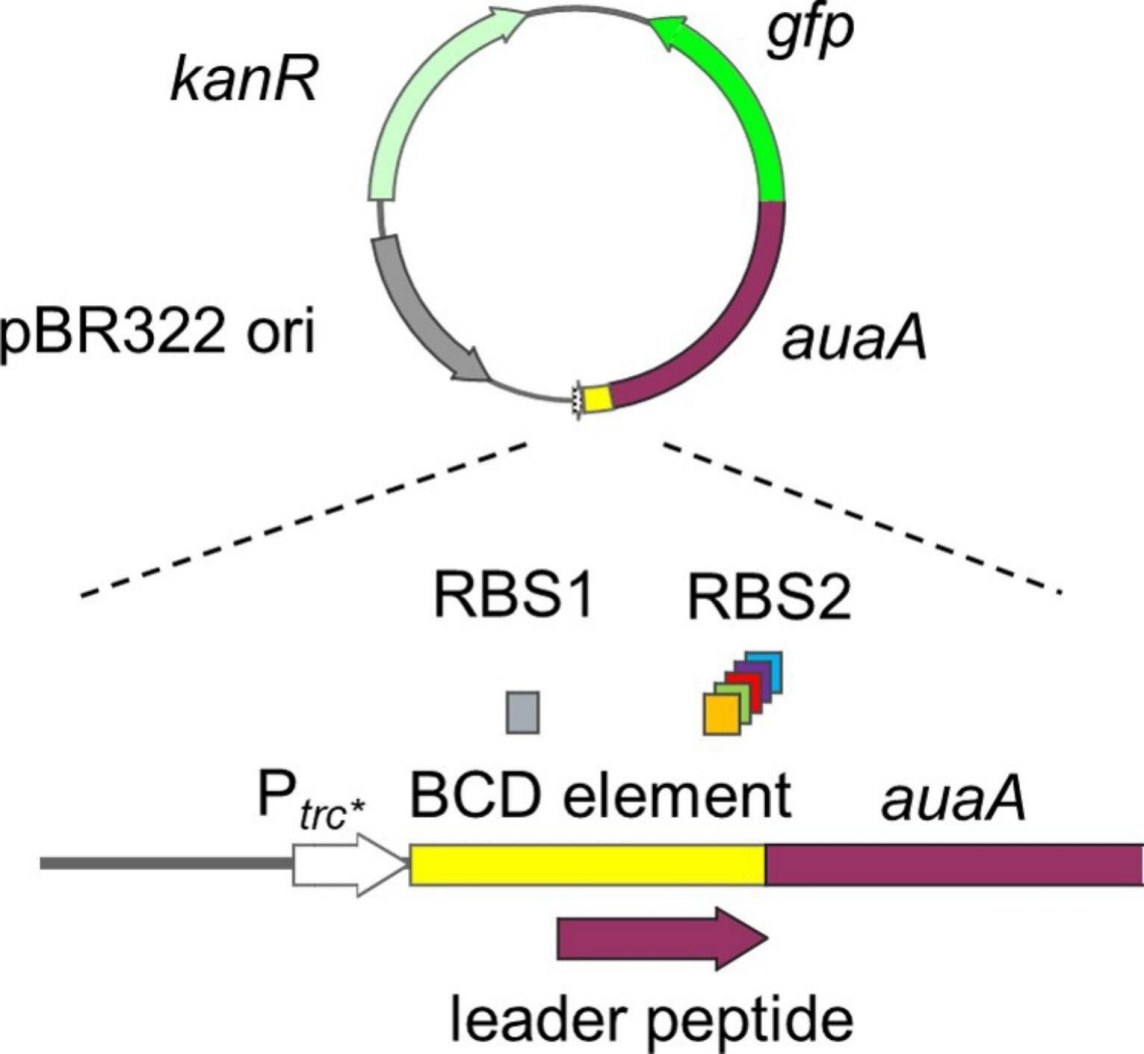
Architecture of the BCD vectors that were used for the expression of *auaA*. The trc* promoter lacks an operator sequence and therefore acts as medium-strength constitutive promoter

In order to generate an *E. coli* strain, which expresses correctly folded AuaA at high level, we screened BCD elements of varying translation initiation strengths. Instead of newly designing these DNA sequences, we utilized the 22 standardized BCDs that had been described and characterized by Mutalik et al. (2013) before. For the assembly of the BCD vectors, we resorted to the method of Claassens et al. (2019), which is based on Golden Gate cloning (Engler et al. 2008). Furthermore, the *auaA* gene was fused to *gfp* to facilitate the quantification of its expression level. C-terminal GFP acts as a folding reporter for membrane proteins. This means that the correct folding and the fluorescence of GFP depend on the effective integration of its fusion partner into a membrane (Drew et al. 2001). The basic architecture of the generated expression constructs is depicted in Fig. 2.

### Screening of the BCD library

**Fig. 3:**
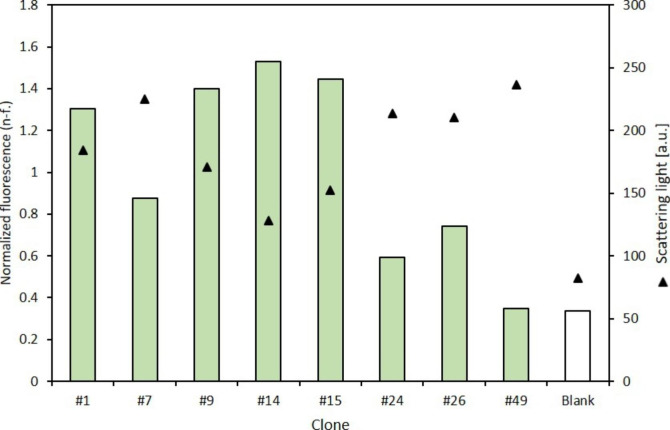
Normalized fluorescence and scattering light measurements of eight arbitrarily selected clones expressing *auaA-gfp* after growing in a 96 well plate for 15 h. The white bar corresponds to a medium blank. The full analysis of all clones can be found in the Supporting Information (Figures S2 and S3)

After the BCD expression vectors were introduced into *E. coli* BL21(DE3), the transformants were picked and subjected to microcultivation in a BioLector system (Kensy et al. 2009). The growth of the cultures was monitored by scattered light measurements. Simultaneously, online fluorescence measurements allowed an estimation of membrane-embedded expression. These analyses revealed considerable variations both in biomass formation and in fluorescence between the cultures, which is exemplarily shown for eight clones in Fig. 3.

**Fig. 4:**
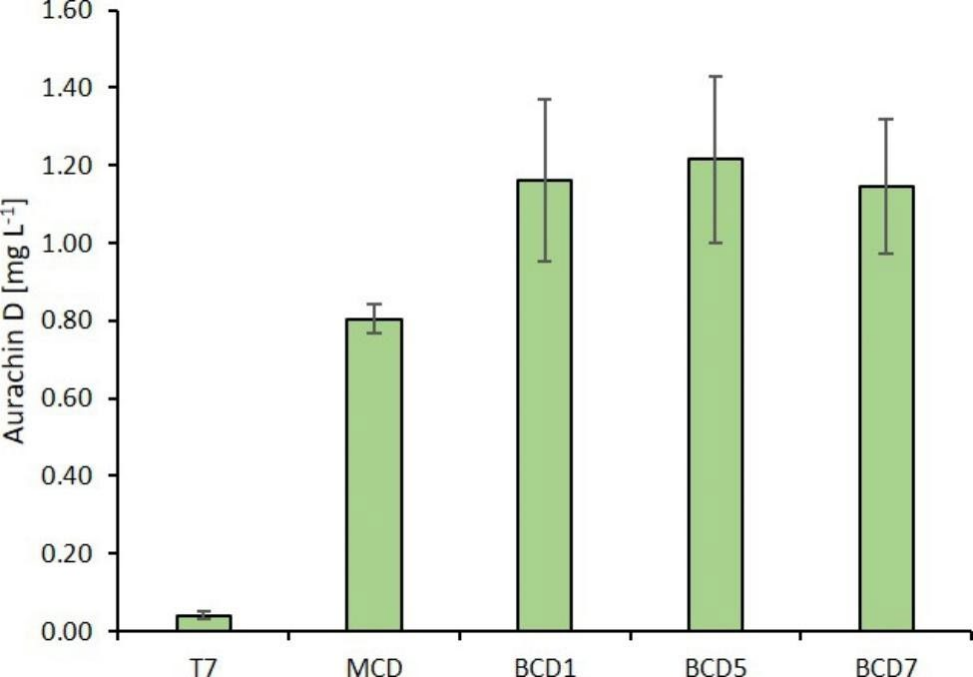
Production of aurachin D in *E. coli* cultures expressing *auaA* under control of the T7 promoter or, alternatively, the trc* promoter using mono- and bicistronic design (MCD, BCD1, BCD5, BCD7)

Following two rounds of screening in the BioLector with different culture volumes (200 µL and 800 µL), nine clones were selected on the basis of their growth characteristics and fluorescence intensities. Sequencing confirmed that the selected clones possessed one out of only three BCD elements, namely BCD1 (3 clones), BCD5 (3 clones), and BCD7 (3 clones). All of these BCD variants were previously reported to mediate a strong translation initiation activity (Mutalik et al. 2013), which seems advantageous for AuaA-GFP production levels. Next, we evaluated the biotransformation of HMQ by each of these clones in production cultures and selected the best producers for the following analyses (Figure S4). The average aurachin D titer of the chosen clones was 1.17 mg L^− 1^ and, thus, almost 29-fold higher compared to the original T7-driven expression system. Further analyses revealed that this improvement was partially due to the replacement of the T7 with the trc* promoter and partially due to the translational coupling architecture (Fig. 4).

### Improvement of the FPP pool in ***E. coli*** expressing ***auaA***

To further increase the aurachin D titer, we then addressed the FPP supply for the biotransformation reaction. In general, the heterologous production of FPP-derived compounds is limited in *E. coli*, because the bacterium maintains only a small pool of this precursor as part of its primary metabolism (Cao et al. 2019). A proven strategy to enhance the metabolic flux to FPP is to introduce the non-native mevalonate pathway into *E. coli* (Rolf et al. 2020). We evaluated this option by co-transforming *E. coli* BL21(DE3) with the BCD7 vector and pJBEI-2997. The latter plasmid harbors the mevalonate pathway genes integrated into a single operon under the control of the lacUV5 promoter (Peralta-Yahya et al. 2011). After preliminary experiments in the BioLector had indicated an IPTG concentration of 0.025 mM to give the best growth results, production cultures of the *E. coli* strain possessing both plasmids, BCD7 and pJBEI-2997, were initiated. These cultures reached an average aurachin D titer of 9.46 ± 0.75 mg L^− 1^, which is more than 237 times higher than the original T7-driven expression system and still an eightfold improvement compared to the exclusive usage of a BCD-based expression strategy.

### Codon optimization of ***auaA***

Another way to improve the expression of a heterologous gene is to adapt its codon usage to the production host. The mevalonate plasmid pJBEI-2997 already contained genes that had been tailored to an expression in *E. coli* (Peralta-Yahya et al. 2011). However, the *auaA* gene originated from a myxobacterium and, hence, its codon usage as well as it G + C content differed significantly from the preferences of *E. coli*. Therefore, codon-optimized versions of the *auaA* genes from the native aurachin producers *S. erecta* Pd e32 and *S. aurantiaca* Sg a15 were designed and synthesized. The expression of the codon-optimized genes, *auaA*_Se and *auaA*_Sa, was subsequently tested using the constructed BCD library. The clones, which gave the highest fluorescence intensities in the BioLector screening (Figures S5–S8), featured other BCD elements than those identified in the previous analysis of the native *auaA* gene.

**Fig. 5:**
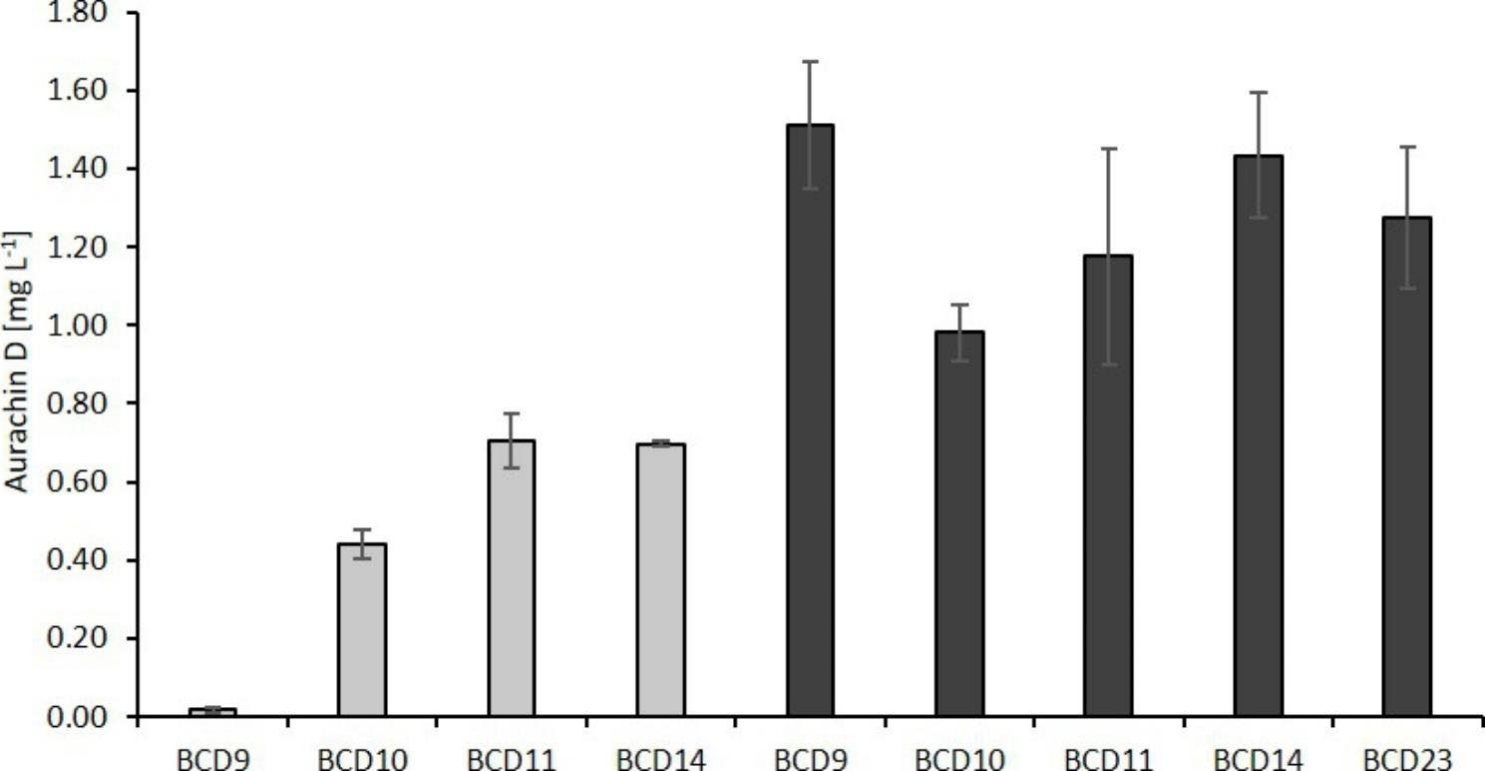
Production of aurachin D in *E. coli* cultures expressing *auaA*_Se (grey bars) and *auaA*_Sa (black bars) under the control of the trc* promoter using different BCD elements

Of the nine selected clones expressing *auaA*_Se, five possessed the BCD10 element, two harbored BCD11, and the remaining two possessed BCD9 or BCD14, respectively. Again, we tested all clones for their capability to biotransform HMQ and selected the best aurachin D producers for a given BCD element (Figure S9). The highest aurachin D titers of 0.70 ± 0.07 mg L^− 1^ and 0.70 ± 0.01 mg L^− 1^ were obtained with clones featuring the BCD11 or the BCD14 element, respectively (Fig. 5). Surprisingly, the clone featuring the BCD9 element reached an aurachin D titer of only 0.02 ± 0.01 mg L^− 1^. Retesting in the BioLector revealed that this clone had also lost its high fluorescence intensity, suggesting a spontaneous mutation in the expression cassette.

Analysis of the nine *auaA*_Sa clones revealed almost the same BCDs as those previously observed in the *auaA*_Se clones, i.e. BCD11 (4x), BCD9 (2x), BCD10 (1x), BCD14 (1x), and BCD23(1x). All clones were again selected based on their aurachin D production (Figure S10). Here, the highest aurachin D titer was determined as 1.51 ± 0.16 mg L^− 1^ for a clone with the BCD9 element (Fig. 5). Strikingly, the BCD sites supporting the highest expression levels in conjunction with *auaA*_Se and *auaA*_Sa are weaker in translation initiation activity than BCD1, BCD5 and BCD7 (Mutalik et al. 2013), which worked best for the expression of the native *auaA* gene. This observation suggests a correlation between the required strength for translation initiation and the codon bias of the expressed gene.

We also analyzed the expression of the mevalonate pathway from pJBEI-2997 on the production of aurachin D in the *auaA*_Se BCD11 and *auaA*_Sa BCD9 clones, respectively. While the *auaA*_Se BCD11 clone reached an aurachin D titer of 8.67 ± 2.52 mg L^− 1^ in the presence of the mevalonate pathway, which is slightly lower than the value obtained with *auaA* BCD7, the combination of *auaA*_Sa and BCD9 resulted in an aurachin D titer of 16.96 ± 0.71 mg L^− 1^. In case of the *auaA* gene from *S. erecta* Pd e32, it can thus be concluded that the codon optimization had either no effect on aurachin D production or that the effect was blurred due to the different BCD elements in the analyzed clones.

## Discussion

Aromatic prenyltransferases catalyze regioselective Friedel-Crafts alkylations of aromatic acceptor molecules with diverse isoprenoid diphosphates (Chen and Abe 2021). Not only are these enzymes involved in the biosynthesis of several bioactive natural products, but also some of their members have been identified as versatile biocatalysts in terms of substrate tolerance and in their ability to form carbon-carbon as well as carbon-heteroatom bonds (Winkelblech et al. 2015). Due to these favourable properties, enzyme-mediated prenylations have gained interest in the field of organic synthesis and for biotechnological applications alike (Wessjohann et al. 2013). While soluble aromatic prenyltransferases are already widely used in these areas, e.g. for the recombinant production of cannabinoids (Zirpel et al. 2017), the membrane-bound representatives are rarely exploited due to the difficulties associated with the expression of these enzymes.

In this study, we explored the heterologous production of a myxobacterial, membrane-bound prenyltransferase in *E. coli*. The corresponding enzyme, AuaA, catalyzes the key transformation in the biosynthesis of aurachin D (Stec et al. 2011), which is used as a tool compound and as a molecular scaffold in drug development (Enomoto et al. 2014; Grauel et al. 2021). In order to achieve a high-level expression of functional AuaA in *E. coli*, we employed an optimized gene expression cassette involving BCD (Mutalik et al. 2013; Sun et al. 2021). This setup was clearly superior in comparison to T7-driven expression. The conversion of the fed substrate HMQ was significantly increased, as evidenced by a 29-fold higher product titer. One prerequisite for this improvement was the identification of an adequate BCD. For this, the screening of a library of translational coupling elements was unavoidable, since it is still not possible to predict the perfect BCD for a given gene. Claassens et al. (2019) previously observed that the overexpression of endogenous and heterologous membrane proteins in *E. coli* yielded the best results when medium-strength BCDs were used. Consistent findings were made in this study only with regard to the expression of the codon-optimized *auaA* genes. In case of the native *auaA* gene, the expression level responded more favourably to BCD variants mediating high translation initiation strengths. Additional studies on different membrane proteins are certainly warranted to clarify the reasons for these differences.

The implementation of the optimized expression cassette led to a considerable increase of the aurachin D titer in the biotransformation experiments, likely mediated through enhanced mRNA stability and translation efficiency. Still, improvements of the whole cell biocatalysis are possible, as exemplified here with the overexpression of the mevalonate pathway. Except for metabolic engineering strategies, which could be pursued to maximize the flux to FPP, it would be also interesting in the future to evaluate different promoters (Neves et al. 2020) or to test the expression of *auaA* in *E. coli* strains with a protein synthesis machinery that was rewired to withstand the toxicity caused by membrane protein overexpression (Gialama et al. 2017; Michou et al. 2020). Since increasing concentrations of the antibiotic aurachin D can be expected to eventually affect the growth of *E. coli* and, hence, to limit the whole cell biocatalysis, preventive measures such as liquid-liquid extractive fermentations must be evaluated as well. In sum, these efforts will provide a microbial cell factory that is not only useful for the production of aurachin D, but will also give facile access to other aurachin-type, farnesylated quinolones.

## Electronic supplementary material

Below is the link to the electronic supplementary material.

**Fig. S1** Linear correlation between the total AUC of aurachin D in HPLC analyses and its concentration
**Fig. S2** Normalized fluorescence and scattering light measurements of all colonies expressing *auaA-gfp* after growing in a 96 well plate for 15 h
**Fig. S3** Normalized fluorescence and scattering light measurements of all colonies expressing *auaA-gfp* after growing in a 48 well plate for 20 h3
**Fig. S4** Production of aurachin D in *E. coli* cultures expressing *auaA*
**Fig. S5** Normalized fluorescence and scattering light measurements of all colonies expressing *auaA_Se-gfp* after growing in a 96 well plate for 15 h.
**Fig. S6** Normalized fluorescence and scattering light measurements of all colonies expressing *auaA_Se-gfp* after growing in a 48 well plate for 20 h.
**Fig. S7** Normalized fluorescence and scattering light measurements of all colonies expressing *auaA_Sa-gfp* after growing in a 96 well plate for 15 h.
**Fig. S8** Normalized fluorescence and scattering light measurements of all colonies expressing *auaA_Sa-gfp* after growing in a 48 well plate for 20 h.
**Fig. S9** Production of aurachin D in *E. coli* cultures expressing *auaA*_Se
**Fig. S10** Production of aurachin D in *E. coli* cultures expressing *auaA_Sa*

## Data Availability

Original data are available upon request. Additional information is provided in the Supplementary Material.
