# Supplementary material for: Biocatalytic production of the antibiotic aurachin D in Escherichia coli
Source: AMB Express. 2022 Nov 3;12:138. doi: 10.1186/s13568-022-01478-8 (PMC9633902; doi:10.1186/s13568-022-01478-8)
Supplement: Supplementary file 1 — Fig. S1 Linear correlation between the total AUC of aurachin D in HPLC analyses and its concentration Fig. S2 Normalized fluorescence and scattering light measurements of all colonies expressing auaA-gfp after growing in a 96 well plate for 15 h Fig. S3 Normalized fluorescence and scattering light measurements of all colonies expressing auaA-gfp after growing in a 48 well plate for 20 h3 Fig. S4 Production of aurachin D in E. coli cultures expressing auaA Fig. S5 Normalized fluorescence and scattering light measurements of all colonies expressing auaA_Se-gfp after growing in a 96 well plate for 15 h. Fig. S6 Normalized fluorescence and scattering light measurements of all colonies expressing auaA_Se-gfp after growing in a 48 well plate for 20 h. Fig. S7 Normalized fluorescence and scattering light measurements of all colonies expressing auaA_Sa-gfp after growing in a 96 well plate for 15 h. Fig. S8 Normalized fluorescence and scattering light measurements of all colonies expressing auaA_Sa-gfp after growing in a 48 well plate for 20 h. Fig. S9 Production of aurachin D in E. coli cultures expressing auaA_Se Fig. S10 Production of aurachin D in E. coli cultures expressing auaA_Sa [file 13568_2022_1478_MOESM1_ESM.pdf]

Biocatalytic Production of the Antibiotic Aurachin D in *Escherichia coli*

Sebastian Kruth,[a] Lina Schibajew,[a] and Markus Nett[a,#]

[a] TU Dortmund University, Department of Biochemical and Chemical Engineering, Dortmund, Germany

#Address correspondence to Markus Nett, markus.nett@tu-dortmund.de

Table of contents

Fig. S1 Linear correlation between the total AUC of aurachin D in HPLC analyses and its concentration 2

Fig. S2 Normalized fluorescence and scattering light measurements of all colonies expressing *auaA*-*gfp* after growing in a 96 well plate for 15 h. 2

Fig. S3 Normalized fluorescence and scattering light measurements of all colonies expressing *auaA*-*gfp* after growing in a 48 well plate for 20 h. 3

Fig. S4 Production of aurachin D in *E. coli* cultures expressing *auaA* 3

Fig. S5 Normalized fluorescence and scattering light measurements of all colonies expressing *auaA*_Se-*gfp* after growing in a 96 well plate for 15 h. 4

Fig. S6 Normalized fluorescence and scattering light measurements of all colonies expressing *auaA*_Se-*gfp* after growing in a 48 well plate for 20 h. 4

Fig. S7 Normalized fluorescence and scattering light measurements of all colonies expressing *auaA*_Sa-*gfp* after growing in a 96 well plate for 15 h. 5

Fig. S8 Normalized fluorescence and scattering light measurements of all colonies expressing *auaA*_Sa-*gfp* after growing in a 48 well plate for 20 h. 5

Fig. S9 Production of aurachin D in *E. coli* cultures expressing *auaA*_Se 6

Fig. S10 Production of aurachin D in *E. coli* cultures expressing *auaA*_Sa 6

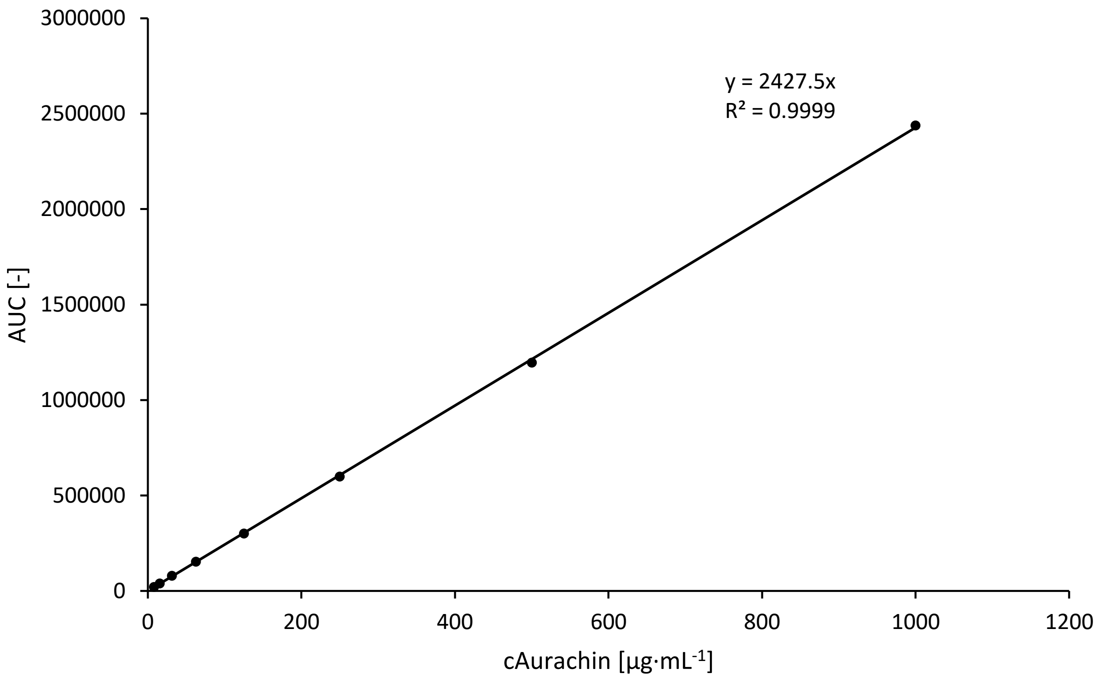

Fig. S1 Linear correlation between the total AUC of aurachin D in HPLC analyses and its concentration. The calibration curve was obtained with commercial aurachin D (AOBIOUS).

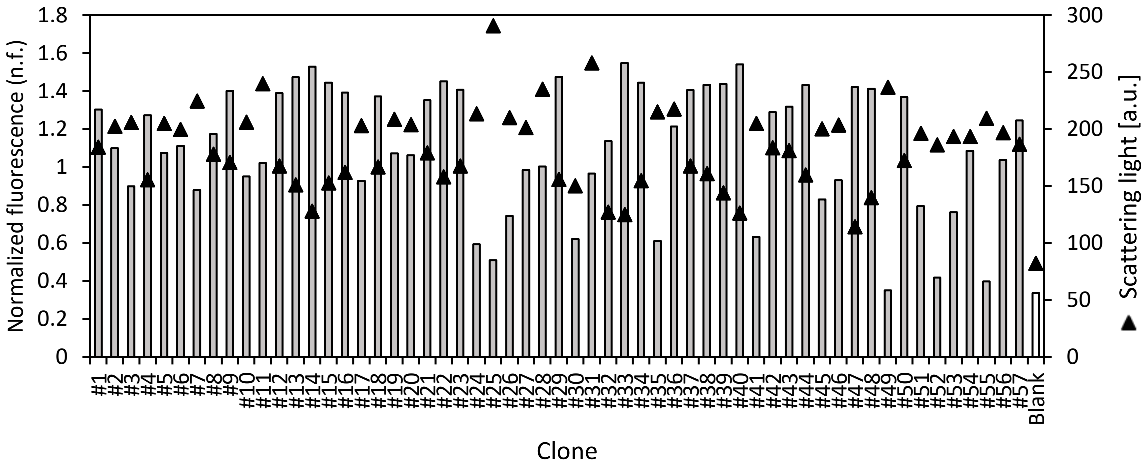

Fig. S2 Normalized fluorescence and scattering light measurements of all colonies expressing *auaA-gfp* after growing in a 96 well plate for 15 h. The white bar corresponds to a medium blank.

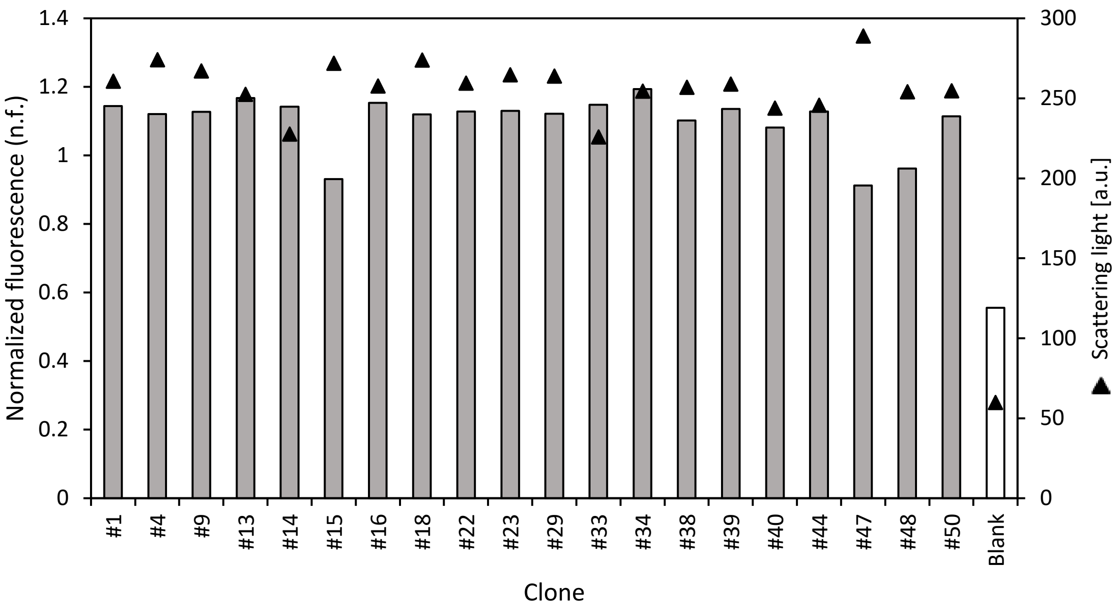

Fig. S3 Normalized fluorescence and scattering light measurements of all colonies expressing *auaA-gfp* after growing in a 48 well plate for 20 h. The white bar corresponds to a medium blank.

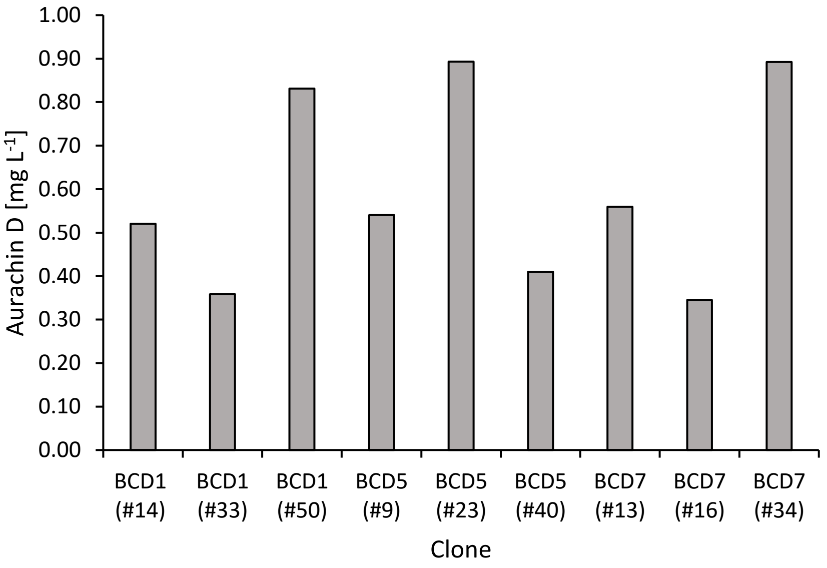

Fig. S4 Production of aurachin D in *E. coli* cultures expressing *auaA* under the control of the *trc** promoter using bicistronic design. For every clone, a single production culture was prepared.

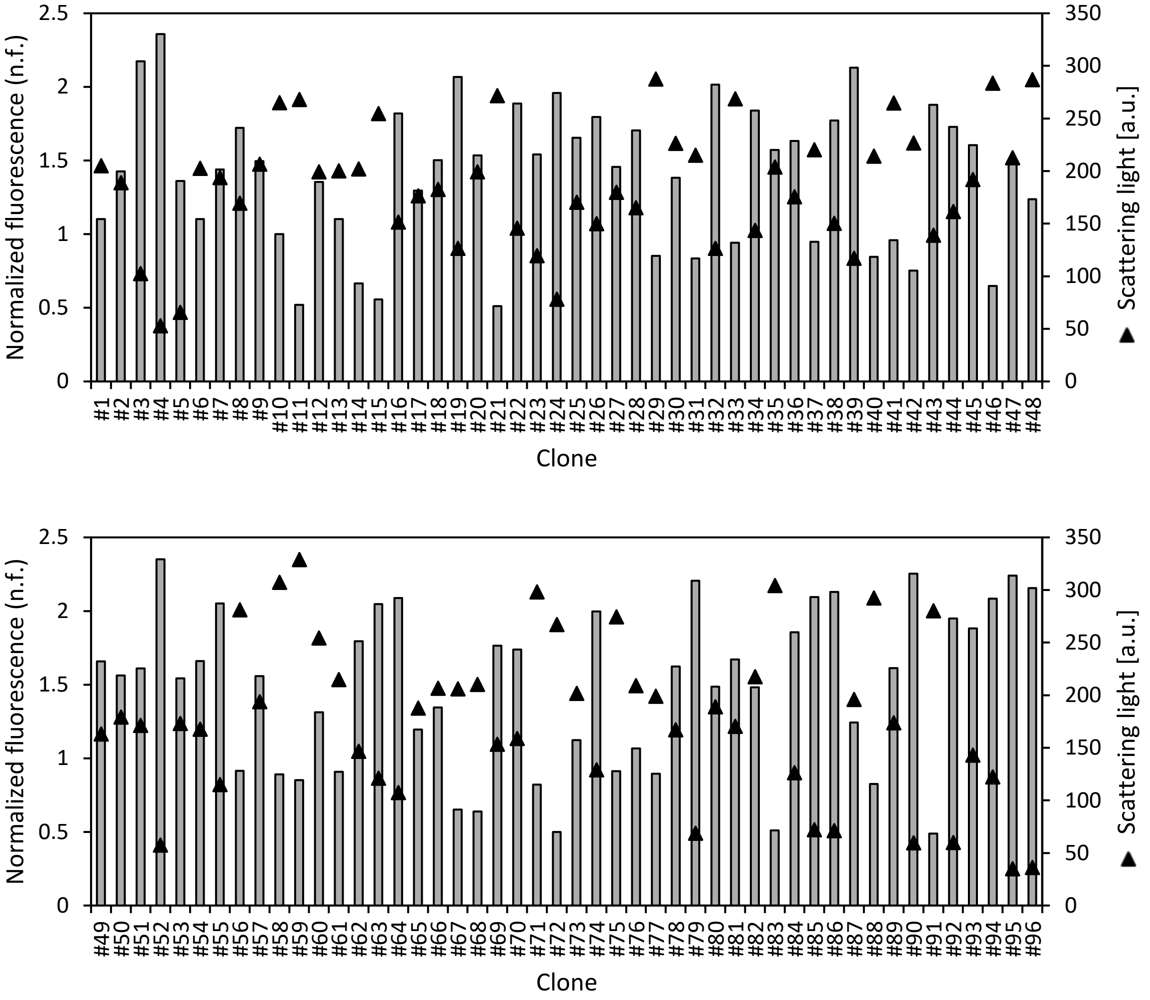

Fig. S5 Normalized fluorescence and scattering light measurements of all colonies expressing *auaA*_Se-*gfp* after growing in a 96 well plate for 15 h.

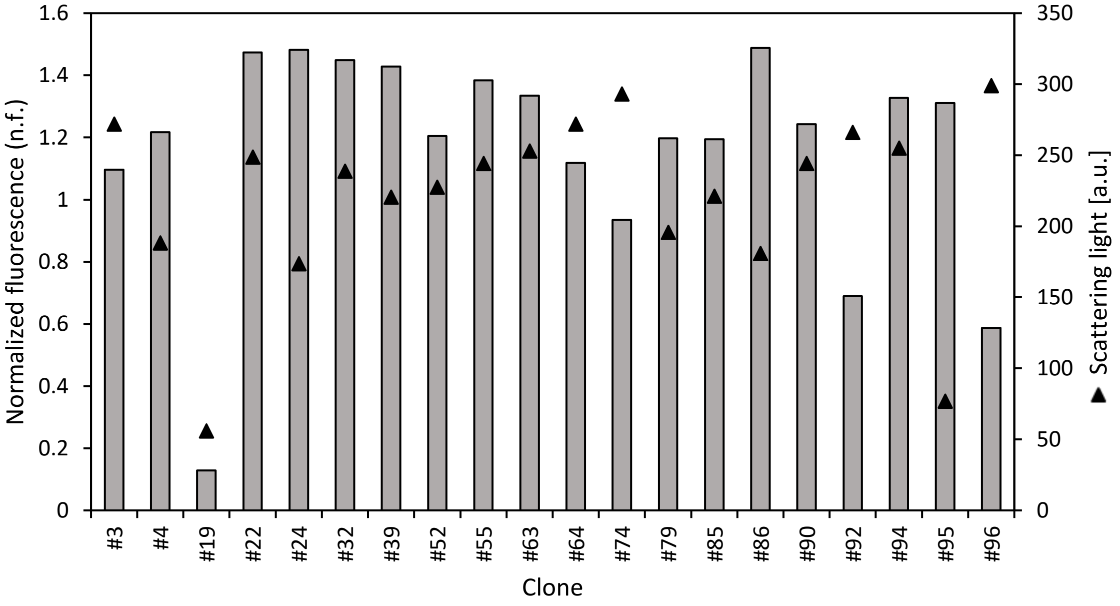

Fig. S6 Normalized fluorescence and scattering light measurements of all colonies expressing *auaA*_Se-*gfp* after growing in a 48 well plate for 20 h.

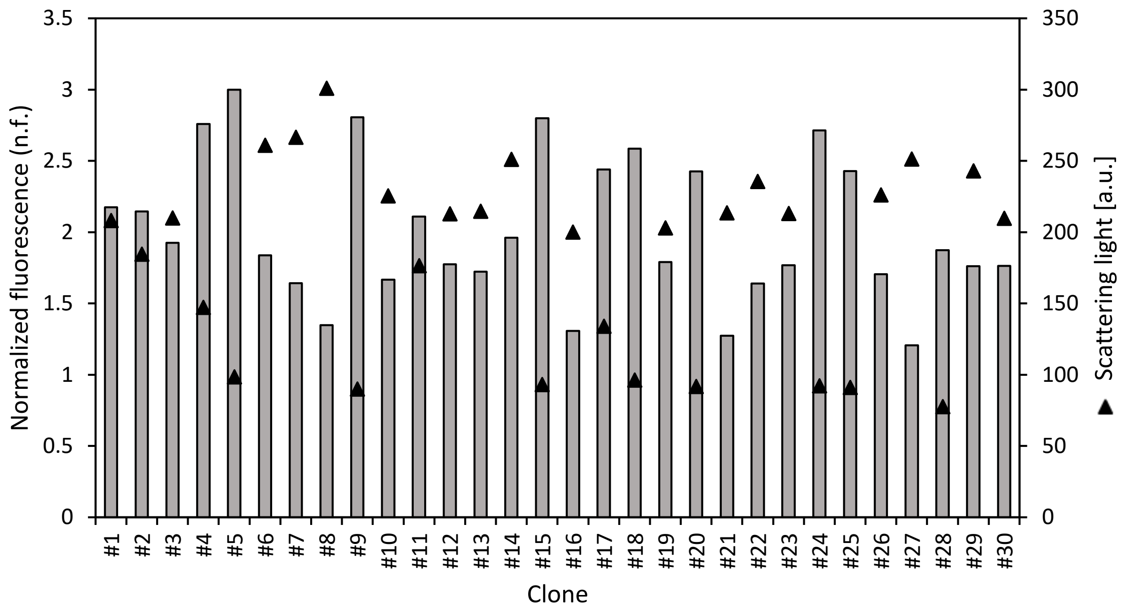

Fig. S7 Normalized fluorescence and scattering light measurements of all colonies expressing *auaA*_Sa-*gfp* after growing in a 96 well plate for 15 h.

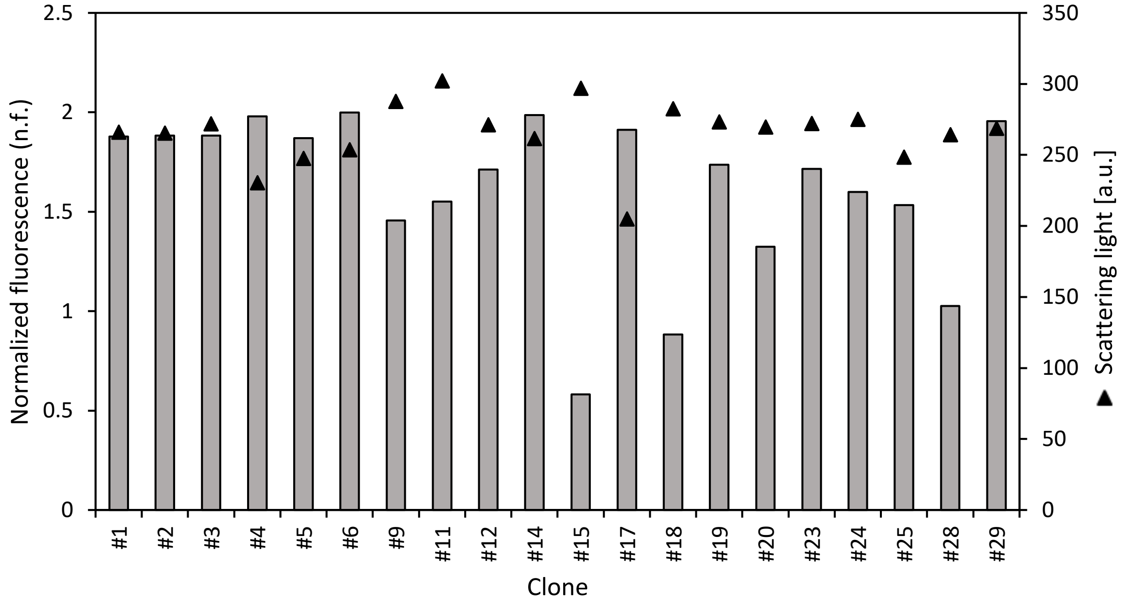

Fig. S8 Normalized fluorescence and scattering light measurements of all colonies expressing *auaA*_Sa-*gfp* after growing in a 48 well plate for 20 h.

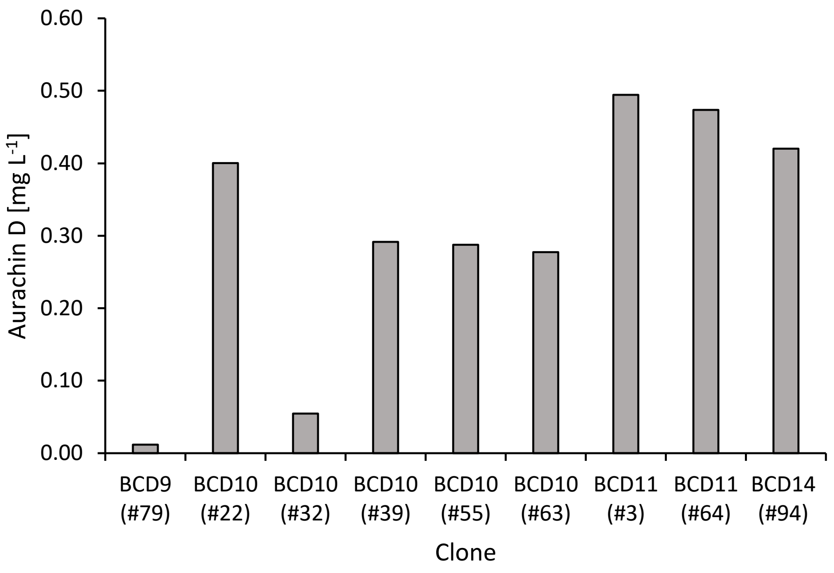

Fig. S9 Production of aurachin D in *E. coli* cultures expressing *auaA*_Se under the control of the *trc** promoter using bicistronic design. For every clone, a single production culture was prepared.

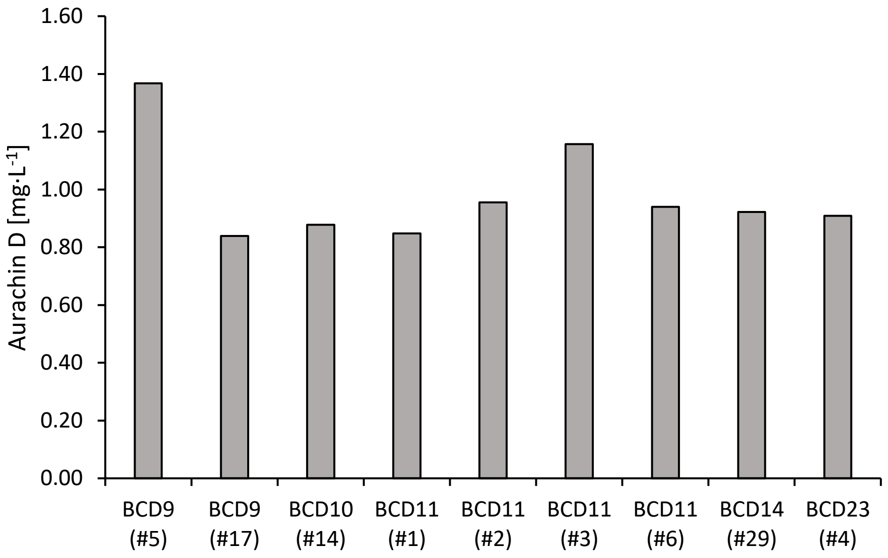

Fig. S10 Production of aurachin D in *E. coli* cultures expressing *auaA*_Sa under the control of the *trc** promoter using bicistronic design. For every clone, a single production culture was prepared.
